# Empowerment interventions, knowledge translation and exchange: perspectives of home care professionals, clients and caregivers

**DOI:** 10.1186/1472-6963-8-177

**Published:** 2008-08-20

**Authors:** Denise St-Cyr Tribble, Frances Gallagher, Linda Bell, Chantal Caron, Pierre Godbout, Jeannette Leblanc, Pascale Morin, Marianne Xhignesse, Louis Voyer, Mélanie Couture

**Affiliations:** 1 École des sciences infirmières, Faculté de médecine et des sciences de la santé Université de Sherbrooke, 3001, 12e Avenue Nord, Sherbrooke, Québec, J1G 4M9, Canada; 2 École de science infirmière, Université de Moncton, Moncton, Nouveau-Brunswick, E1A 3E9, Canada; 3 Département de psychologie, Université de Sherbrooke, 2500, boul. Université, Sherbrooke, Québec, J1K 2R1, Canada; 4 Centre de santé et de services sociaux-Institut universitaire de gériatrie de Sherbrooke, 500, rue Murray, Sherbrooke, Québec, J1G 2K6, Canada; 5 Département de médecine de famille, Faculté de médecine et des sciences de la santé Université de Sherbrooke, 3001, 12e Avenue Nord, Sherbrooke, Québec, J1G 4M9, Canada

## Abstract

**Background:**

Few studies have examined empowerment interventions as they actually unfold in home care in the context of chronic health problems. This study aims to document the empowerment process as it plays out in interventions with adults receiving home care services.

**Methods/design:**

The qualitative design chosen is a fourth generation evaluation combined with case studies. A home care team of a health and social services center situated in the Eastern Townships (Québec, Canada) will be involved at every step in the study. A sample will be formed of 15 health care professionals and 30 of their home care clients and caregiver. Semi-structured interviews, observations of home care interventions and socio-demographic questionnaires will be used to collect the data. Nine instruments used by the team in prior studies will be adapted and reviewed. A personal log will document the observers' perspectives in order to foster objectivity and the focus on the intervention. The in-depth qualitative analysis of the data will illustrate profiles of enabling interventions and individual empowerment.

**Discussion:**

The ongoing process to transform the health care and social services network creates a growing need to examine intervention practices of health care professionals working with clients receiving home care services. This study will provide the opportunity to examine how the intervention process plays out in real-life situations and how health care professionals, clients and caregivers experience it. The intervention process and individual empowerment examined in this study will enhance the growing body of knowledge about empowerment.

## Background

This study is part of a process to transform health care practices with the aim of strengthening empowerment of clients with chronic health problems. To achieve this objective, an in-depth analysis of home care interventions will be undertaken using an evolving model of empowerment interventions (enabling interventions). The intervention indicators were derived from the findings of previous studies [1-6].

This study is concept-oriented and empirical in nature and will help to further refine interventions supporting empowerment. It is in line with the reform of the Québec health care system that calls for a transition of services from health care facilities to programs that support people in their living environment. Along with this relocation of services, there is a political debate about how important it is for individuals and their families, caregivers and communities to take responsibility for their own health and the necessary steps to improve it [7,8] This stance implies a modification of professional practices to support self-care. However, interventions supporting empowerment are not well documented, despite the aging of the population and the growing number of home care services following after a short hospital stay [9]. The CSSS is the local authority at the heart of the health and social services network; it provides primary care services to the local population and ensures their accessibility, continuity and quality. It is essential that this organization use scientific knowledge to develop and deliver preventive and primary care adapted to the different needs of the population by supporting self-care initiatives and promoting autonomy.

### Statement of the problem

The clients targeted in this study are adults with chronic health problems who receive home care services. The Ministère de la Santé et Services sociaux du Québec [9] identifies chronic diseases as the most important health problem of the century: "Chronic diseases develop slowly, persist over time, are often incurable and result in a disability and, above all, they claim many victims. Four chronic diseases are responsible for more than 70% of deaths in Québec every year. They are, in order of importance, cancers, cardiovascular diseases (CVD), respiratory diseases and diabetes." In the Eastern Townships alone, 51.4% of the population suffers from at least one chronic health problem and 19.7% live with activity limitations [10]. The main health problems found in the Eastern Townships and particularly the City of Sherbrooke are cardiovascular and respiratory diseases, diabetes and breast cancer [11,12]. These health problems often lead to long-term activity limitations requiring home care services [12]. Moreover, family members (caregivers) frequently help with the care of individuals suffering from one or many chronic diseases and with the daily management required by these types of diseases. It was estimated that in 1996, 2.8 million Canadians aged 18 years and older provided help for a person with long-term limitations [13]. In order to improve the health status of this clientele and family support, Québec's homecare services have adopted a framework that advocates interventions promoting empowerment. These interventions are aimed at enhancing clients' potential to learn and use the tools they need to live as independently as possible and to maintain or improve their own and their family's quality of life [14,15]. The interventions promoting empowerment can be conceptualized as being part of a process that enables the development of the capacity to choose, to make sound decisions and to act according to one's preference [16]. This in turn, according to Dunst and Trivette [17], increases the person's control over his or her life and is associated with various cognitive attributes, such as intrinsic motivation, self-efficacy and self-awareness. According to this perspective, it is possible for a person to learn to be more independent, to adopt healthier lifestyles and to relate better to his or her social environment and network.

Research has shown that professional home health care providers frequently mention the use of interventions promoting self-care when describing their work. Their description of the interventions indicates that their professional expertise is employed within a collaborative relationship [1,18]. More research is needed to examine how empowerment interventions unfold in real-life practice settings [2]. This is particularly important since the home care sector seems to assume that their programs are empowering [7,9] although there is little empirical evidence supporting this claim. With the transition of health care services to the community and the ever-increasing emphasis on empowerment, the time seems right for a study observing interventions with clients suffering from chronic health problems who require home care services [4,5].

In the context of this study and based on prior work by members of the research team, the term *enabling interventions *refer to professional interventions that promote empowerment. The terms *individual empowerment *refers to the impact of these interventions on the persons receiving them [1,2,4-6,19,20].

Empowerment research is first and foremost theoretical in nature and there is very little empirical information on the full extent of the phenomenon. The available data are predominantly from studies examining the process of community empowerment with groups of individuals representing extreme cases of isolation, such as persons with disabilities and persons who are economically underprivileged [21-23]. Empowerment has also been studied in reference to closely related concepts such as compliance, coping and self-care. However, these concepts are limited compared to the more comprehensive approach associated with the phenomenon of empowerment. In fact, these closely related concepts could be part of a larger empowerment construct [6]. Despite these limitations, prior studies have identified many essential attributes of enabling interventions for communities and individuals.

Among authors who have attempted to define the attributes of empowerment interventions, Gibson [24] states that this type of intervention focuses more on the individual's strengths, rights and capabilities than on his or her shortfalls. This approach implies that people are seen not as powerless victims but as being able to identify solutions for their well-being [25]. In addition, enabling interventions must help people develop their "self-solutions" [26]. Cardone and Gilkerson [27] and Dunst and Trivette [17] maintain that considering persons as possessing knowledge rather than as recipients of professional knowledge enhances the use of their own competencies. In a study with families living in extreme poverty, Ouellet et al. [22] observed certain characteristics that influence enabling interventions, such as follow-up duration and intensity, the availability of the health care professional and intervention flexibility. Zerwekh [28] states that interventions promoting empowerment require a balance between the care recipient's initiatives and those of the health care professional. Strategies that foster a person's empowering capabilities include: 1) listening to concerns; 2) supporting awareness of alternatives; and 3) broadening possibilities. For their part, Paul et al. [1] maintain that enabling interventions are founded on the latitude given to a person within the intervention and take into account the individual's experiences and perceptions of his or her reality. In a prior study, five dimensions that comprise enabling interventions were identified. They are interventions that: 1) develop and maintain a therapeutic relationship; 2) are based on a person's point of view and strengths; 3) encourage and support the decision-making process; 4) help to broaden possibilities; and 5) facilitate the learning experience [2]. Some studies show that in order to facilitate empowerment, an egalitarian relationship must be established with the person having difficulties [22,28,29], a relationship based on collaboration and that is respectful of the person's experience and ability to seek solutions to his or her problems [30]. The relationship also implies that the person is able to define his or her own needs [2]. To date, very few studies have described the context in which professional interventions unfold with people needing home care services.

### Factors associated with empowerment interventions

Interventions that encourage empowerment are influenced by the community health care professionals' perceptions and modes of understanding and their ability to interact with the people involved [17,21]. According to Labonté [31], empowerment exists as a conceptual lens through which professional practices can be revaluated. Some factors might restrain the application of enabling interventions by health care professionals and compromise their enabling effect: 1) a vision of the situation focused primarily on problems and the person's deficits [24]; 2) a misunderstanding of the person's own resources [32]; 3) a stereotypical attitude towards a person's abilities and limitations influenced by social class and family structure [33]; 4) a tendency to consider the professional point of view as reliable and to disregard that of the care recipient; 5) different and sometimes opposing views on the definition of power between the help-giver and the help-seeker and on the distribution of power within the intervention [34,35]; and 6) the professional's experience and feelings of self-efficacy related to the intervention [2]. When examining interactions between professionals and their clients, it is imperative to take into account clients' perceptions of their own abilities, limitations and needs as well as their vision of the interactional process.

### Individual empowerment as the outcome of enabling interventions

Individual empowerment reinforces various behaviours and encourages the person to take more control over events and important situations in his or her life [17]. Drolet [36] mentions that individual empowerment contributes to the development of problem-solving skills and increases self-esteem and self-efficacy. St-Cyr-Tribble et al. [2] identified several indicators of individual empowerment. These indicators are: 1) awareness of one's life situation, own strengths and needs; 2) increase in self-esteem; 3) decrease in negative feelings; 4) well informed decision-making; 5) learning and developing skills; 6) taking action; 7) developing relationships with the social support environment and network; and 8) improvement in living conditions. These dimensions of empowerment correspond to what Rissel [35] called *psychological or individual empowerment *in his community empowerment model. Empowerment is a long-term process of change [37,38], a dynamic phenomenon comprising a number of steps or phases, but there is no consensus between authors concerning the whole process. Usually the process starts with an awareness phase and ends with an action phase, be it individual or collective in nature [35]. The awareness phase is often generated by a crisis or contextual change [22,37,39]. Studies that have explored aspects of individual empowerment rarely did so in the context of home care services. Furthermore, most results are based on representation and not on observations of the intervention process itself. Therefore, there is almost no information concerning indicators associated with the efficacy of these interventions.

### Elements associated with the process of individual empowerment

Some authors believe there is a link between personal resources and the individual empowerment process [19,38]. For example, having good solid values, using available resources, being responsible, possessing internal strength, being able to improve one's self-confidence or desiring a better future are all linked to the empowerment process [21]. The latter two elements have also been noted in research done by St-Cyr Tribble et al. [2]. Living conditions such as poverty and the quality of the social environment, and especially social support, can hinder the individual empowerment process and maintain helplessness [21,40].

### Efficacy of empowerment interventions

A few evaluative studies on the efficacy of enabling interventions have been carried out in the field of family services. These studies mostly used data from interviews with health care professionals and their clients to gather information on the interventions and their outcomes. To our knowledge, only Gibson [41] has used participant observations to examine the process of individual empowerment in mothers with a child suffering from a chronic disease. Gibson's study did not focus on the intervention process *per se*. Because the intervention process can be seen as being in constant flux between the clients' individual characteristics and their particular contexts [42], it is necessary to study empowerment intervention in all its complexity. The enabling intervention should be examined in its natural context and should take into account the client's point of view. It is then possible to determine the indicators of these interventions in terms of their agreement with the literature, on the one hand, and with health care professionals' and clients' perspectives on the other.

### Purpose and objectives of the study

The principal aim of the study is to document the empowerment process (empowerment interventions and individual empowerment) of adults receiving home care services and their caregivers. More specifically, the objectives of the study are to: 1) describe the modes of actualization of enabling interventions in the home care setting; 2) describe the individual empowerment process of home care clients; 3) obtain the caregivers' (professionals and lay persons) views on empowerment interventions, the process of individual empowerment and the integration of knowledge; 4) examine the relationship between empowerment interventions and the process of individual empowerment of home care clients; 5) describe the contextual elements facilitating the implementation of empowerment interventions and individual empowerment; and 6) document the transfer and integration of knowledge between the research team, health care professionals, lay persons, caregivers and home care clients of the community health division of the CSSS-IUG of Sherbrooke.

### Theoretical framework

The concept of empowerment is studied from an individual perspective even though the phenomenon comprises a collective component [35,43]. From an individual perspective, empowerment is a social process whereby the acquisition of skills by the person to satisfy his or her needs, resolve his or her problems and mobilize the necessary resources to take control over his or her life is recognized, supported and valued [34].

## Methods/design

### Research design

A mixed qualitative design combining a fourth generation evaluation and case studies [44,45]. The fourth generation evaluation serves as a collaborative approach that encourages dialogue between the actors in the ongoing research. A steering committee composed of investigators, "on-site" co-investigators and administrators has been created and will be responsible for the planning and progress of the study, coordinating communications and maintaining interactions with the practice setting as well as integrating the research findings. An on-site follow-up committee composed of health care professionals will help define contact modalities with clients, and contribute to the validation of research evaluation tools, revision of the analyzed data and development of clinical tools. The case study approach will contribute to the understanding of how enabling interventions influence the individual empowerment process for clients and their caregivers receiving home care services.

### Participants

A theoretical sample [46] will comprise 15 health care professionals (nurse, psychosocial professional, respiratory care therapist, etc.) working in the home care program of a CSSS and 30 individuals aged between 18 to 64 years who have chronic health problems such as diabetes, cardiovascular and respiratory disease and are receiving home care services. The age range was chosen to allow maximum variability when studying the concept of empowerment with adults. Caregivers will also be asked to participate in an interview, for a possible maximum of 30. Caregivers are defined as individuals providing long term care to a member of their family, a friend, or a neighbour suffering from a chronic disease [47]. The clients will designate a person they consider as a caregiver among their social network. Participants' inclusion criteria are presented in Table 1. The decisions regarding the sample size of this theoretical and purposeful sample study are based on qualitative research's criteria [4,46]. One of the principal criteria is that the amount and the depth of the data collected (n = 4 methods) is more important to achieve the aims of a qualitative study than the number of participants. Another criteria is saying that triangulation of multiple methods design (in this study: evaluative and cases study), types of participants (home care professionals, n = 15; clients, n = 30; caregivers, n = 30), numerous research tools (n = 9), are all insuring the rigor of the process and providing strong substantiation of constructs. Another reason has been influencing the decision making on the sample size. This study on empowerment intervention has been inspired from the results obtained and tools elaborated in other studies on the phenomenon that were conducted by the present group of investigators, thus giving room to methodological experience supporting the sample choice [2-6,14,20].

**Table 1:** Inclusion criteria

Inclusion criteria for health care professionals	Inclusion criteria for home care services clients	Inclusion criteria for caregivers
▪ Working in community home care services ▪ Have held a position or assigned to a replacement as a nurse, psychosocial professional, respiratory care therapist for the last 6 months	▪ Aged between 18 and 64 ▪ Residing in the Eastern Townships ▪ Receiving services at home or in a nursing home for a chronic disease ▪ Receiving five home visits during the study period ▪ Not being in terminal phase ▪ Understanding French and having sufficient cognitive resources to participate in an interview ▪ Accepting to the presence of an observer, allowing the interaction with a professional to be audio taped	▪ Residing in the Eastern Townships ▪ Understanding French and have sufficient cognitive resources to participate in an interview ▪ Agreeing to the presence of observer, allowing the interaction with the professional to be audio taped

### Data collection

**Four methods **of data collection will be used: direct observations of home care visits, semi-structured interviews, focus groups and socio-demographic questionnaires (see Table 2). A non-participatory direct observation will provide *in situ *data during the intervention process [48] and shed new light on the integration of empowerment interventions in professional practice and provide evidence regarding the client's individual empowerment process. The strategy used to select cases for the observation of home visits is an adaptation of the critical incident and case study method [49,50]. Participating health care professionals will intentionally select two cases from their caseloads.

**Table 2:** Data collection methods for the study variables

	Variables			
	
Data collection method	Enabling interventions	Individual empowerment	Contextual elements	Knowledge transfer and exchanges
Direct observation	X	X		X
Focus group	X	X	X	X
Semi-structured interview	X	X		X
Sociodemographic questionnaires			X	

#### Nine tools will be used to collect data

During home care visits, field data will be recorded on a pre-established observational protocol by an observer, and interactions between the health care professional and client will be tape-recorded. The **observational protocol is designed according to **the work of Miles and Huberman [49] and Godbout [4]. It includes broad categories based on the research objectives and a framework of empowerment interventions (see Table 3). The protocol contents will be validated by health care professionals, investigators and other content experts associated with the research project and be pre-tested during a home care visit. In addition to the observational protocol and the recording of interactions, the observer will keep a **personal log **to document his or her perspective and reflections about what is being observed. For each home care case, a tape-recorded semi-structured interview will be carried out with the client and his or her caregiver to gain their perspectives on empowerment interventions, the individual empowerment process and knowledge integration.

**Table 3:** Categories of empowerment practices (attitudes and interventions)

1. Contributing to the therapeutic relationship
▪ Listens actively
▪ Shows interest
▪ Acts with compassion, warmth, honesty, respect and empathy
▪ Uses appropriate, easy to understand and vivid language, uses humour when needed
2. Building on the person's point of view and strengths
▪ Encourages the expression of expectations, needs, hopes, questions, objectives, difficulties...
▪ Encourages the identification of own strengths, prior solutions used
▪ Gives the person sufficient time to express himself or herself
▪ Identifies and values the person's strengths (expertise, solutions, results obtained, etc.)
▪ Expresses his or her availability and maintains continuity of care

3. Encouraging and supporting the decision-making process
▪ Invites the person to decide which needs or problems will be the focus of the intervention, the objectives to be pursued, whether or not to accept the help offered and the solutions and resources proposed
▪ Provides the necessary information for decision-making, accepts the decision taken and adapts the intervention when needed
▪ Adjusts to each person's rhythm

4. Helping to broaden the person's possibilities
▪ Informs
▪ Counsels
▪ Reflects
▪ Normalizes
▪ Reframes
▪ Invites the person to question himself or herself
▪ Facilitates access to diverse resources

5. Facilitating the learning experience
▪ Facilitates attempts to try new ways of doing things or change attitudes
▪ Teaches by using real situations and examples

An **interview guide **with open-ended questions derived from indicators of the individual empowerment process will be used with clients and their caregivers (see Table 4). Another interview guide will also be developed to collect health care professionals' views on their empowerment interventions. This second **interview guide **will be based on results obtained in prior research (see Table 3). Because of the reflective nature of this study, the interview with health care professionals will also probe their views regarding the research process itself, and especially what they have learned or gained from their participation. This will assess the knowledge transfer component of this study between the research team and the health care professionals. A **focus group **conducted with an **interview guide **will give the home care services team the opportunity to share their views about enabling interventions and individual empowerment. This focus group will encompass a larger group of health care professionals in order to obtain data that complement the observational and interview data already obtained. **Socio-demographic questionnaires **based on the Québec Health Survey [51] and those used by the authors in a previous study [2] will be used to record socio-demographic data with each type of participant (health care professionals, clients, caregivers).

**Table 4:** Indicators associated with the individual empowerment process

▪ Reflection and awareness of one's own situation, strengths and needs
▪ Development of self-esteem
▪ Decrease in negative emotions such as stress, anxiety and sorrow
▪ Enlightened decision-making consistent with expectations and needs
▪ Learning and developing communication, social and other skills
▪ Taking action by trying new ways of doing things
▪ Developing relationships with one's support network and community resources
▪ Improving their life conditions

### Data analysis

The observational data (including the field notes and the audiotape transcripts of interactions during the home care visit) and the interview data (individual interviews and focus groups) will be analyzed using a qualitative content analysis approach based on the work of Miles and Huberman [49]. An analysis framework developed previously by St-Cyr Tibble et al. [2] will be adapted and enhanced to conform to the present research objectives by the research team and the members of different committees. Also, discussions with members of the committees will serve as a means to validate the results of the analysis from a clinical and scientific standpoint. NVivo software will be used to manage the qualitative data. The research team will perform the initial coding of the data. Subsequently, 30% of the transcript material will be reviewed by experts recruited from members of the follow-up committee to confirm the results of the categorization. A descriptive analysis of the socio-demographic data will be done to obtain profiles of the clients, caregivers and health professionals. These results, combined with those of the other analyses, will enhance our understanding of different factors potentially having an impact on the empowerment process (enabling interventions, individual empowerment and knowledge transfer).

The conceptualization of empowerment in the current study is based on a review of the literature [24,28,30,35,52,53], and on the results of several empowerment studies conducted with parents of young children, women facing premature labour, adults suffering from chronic diseases, and with health care professionals working with these clienteles [1,2,4,5,19]. It is therefore conceptualized that empowerment interventions contributing to individual empowerment consist of five categories of practices (see Table 3). Table 4 outlines the indicators of individual empowerment for clients and their caregivers.

### Research procedure and timeline

The study will proceed in four stages.

**1. Preparatory procedures for data collection **(June 2006 – October 2006)

▪ Contact home care sector administrators and staff to discuss the project and take their views into account

▪ Create management and follow-up committees

▪ Develop research tools based on empirical results from previous studies

**2. Sampling procedures and data collection **(September 2006 – October 2008)

▪ Selection of a sample of health care professionals, home care services clients, caregivers

▪ Observation of home care visit for each of the selected cases

▪ Semi-structured interviews with all participants

▪ Focus group with the home health care professionals to gather additional information

▪ Discussions with the follow-up committee

**3. Data analysis **(concomitant with data collection) (March 2007 – December 2008)

▪ Transcription of the interviews

▪ Content analysis

▪ Discussions with the follow-up committee, the professionals and the co-investigators

▪ Writing the first version of the research report

▪ Presentation of the preliminary results

**4. Publication, knowledge transfer and exchanges **(December 2008 – December 2009)

▪ Creation of a scientific committee comprising co-investigators and health care professionals to organize symposiums

▪ Organization of two workshops for knowledge transfer and exchanges (by the scientific committee in collaboration with the follow-up committee) for CSSS health care workers

▪ Organization of two symposiums on the empowerment process and the results of the study pertaining to the intervention, the family and caregivers

▪ Writing the final version of the report

▪ Dissemination of the results.

### Strategies for knowledge translation and exchanges

To our knowledge, very few studies have explored knowledge transfer as a phenomenon linked to the two important dimensions of empowerment, enabling interventions and individual empowerment. This is surprising considering, on the one hand, that enabling interventions are comprised of actions aimed at enhancing learning and personal growth and, on the other, that manifestations of individual empowerment integrate different forms of knowledge (knowing, know-how, ways of being, including the development of social competencies) and action-taking. It is therefore important for health care professionals to be able to transfer their knowledge effectively to home care recipients in order to support their self-care capabilities and enhance their individual empowerment.

The study design already includes an ongoing knowledge translation and exchanges (KTE) component. Different KTE activities are planned targeting internal and external audiences (scientific breaks and lunches in the workplace, research days, regional, provincial and international symposiums). Articles will be published in professional and scientific journals during and after the research project. Results will be on different Websites. The Canadian Institutes of Health Research [54] state that: "Partnerships are at the heart of all KT activity. Effective KT is underpinned by effective exchanges between researchers and users – exchanges premised on meaningful interaction with intent to appropriate use of the latest and most relevant research in decision-making." It is therefore essential that health care professionals and clients are encouraged to acquire and use new knowledge and that health care institutions support this by creating means for KTE activities that are fuelled by the latest research findings on the process of individual empowerment. An *ad hoc *KTE committee involving practitioners, researchers and decision makers will be in charge of identifying and organizing these activities.

### Ethical considerations

This study has been approved by the Ethics Committees for Research of the CSSS-IUGS and the Eastern Townships institutions that provide services to the community (2006–11). The presence of an observer during the home care visit could represent a source of discomfort for participants. Measures will be taken to protect the integrity and privacy of the clients, health care professionals and caregivers. All the participants: professionals, clients and caregivers will be signing an informed consent prior to their enrolment in the study.

## Discussion

With the ongoing process to transform the health care and social services network, there is a growing need to examine intervention practices of health care professionals working with clients with chronic health problems who receive home care services [55]. This study will provide the opportunity to examine how the intervention process plays out in real-life situations and how health care professionals, clients and caregivers experience it. The dynamic nature of the intervention process and individual empowerment examined in this study will enhance the growing body of knowledge about empowerment. Many health care professionals are taught to act as experts with clients. Promoting self-care initiatives by relying on a person's strengths and supporting his or her progress towards this goal is not a simple task. Studies like this one are needed to understand the processes involved. The use of a reflective approach to study enabling interventions and individual empowerment is therefore an innovative aspect of this research. It should provide cues on how to better support health care professionals in their efforts to adapt and transform their practices so they can guide their clients towards greater autonomy. Another innovative aspect of this study is the use of direct observations of real-life home care visits. This should refine and reinforce our evolving model of empowerment interventions, which is mostly derived from interview data.

## Competing interests

The authors declare that they have no competing interests.

## Authors' contributions

All authors approved the final manuscript and are willing to take the responsibility for appropriate portions of the content.

## Pre-publication history

The pre-publication history for this paper can be accessed here:


